# Supplementary material for: Synthesis and Evaluation of Thermoresponsive Boron-Containing Poly(N-isopropylacrylamide) Diblock Copolymers for Self-Assembling Nanomicellar Boron Carriers
Source: Polymers (Basel). 2018 Dec 29;11(1):42. doi: 10.3390/polym11010042 (PMC6401969; doi:10.3390/polym11010042)
Supplement: Supplementary file 1 [file polymers-11-00042-s001.pdf]

Supplementary Information

Synthesis and Evaluation of Thermoresponsive Boron-Containing Poly(N-isopropylacrylamide) Diblock Copolymers for Self-Assembling Nanomicellar Boron Carriers

Shuichiro Yoneoka [1], *Ki Chul Park* [1], *Yasuhiro Nakagawa* [2,3,4,5], *Mitsuhiro Ebara* [2,3,6],

and Takehiko Tsukahara [1 †]

[1] *Laboratory for Advanced Nuclear Energy, Tokyo Institute of Technology, 2-12-1-N1-6, Ookayama, Meguro-ku, Tokyo 152-8550 Japan*

[2] *International Center for Materials Nanoarchitectonics (WPI-MANA), National Institute for Materials Science (NIMS), 1-1 Namiki, Tsukuba, Ibaraki 305-0044, Japan*

[3] *Graduate School of Pure and Applied Science, University of Tsukuba, 1-1-1 Tennodai, Tsukuba, Ibaraki 305-8577, Japan*

[4] *Graduate School of Engineering, The University of Tokyo, 7-3-1 Hongo, Bunkyo-ku, Tokyo 113-8656, Japan*

[5] *Innovation Center of NanoMedicine, Kawasaki Institute of Industrial Promotion, 3-25-14, Tonomachi, Kawasaki-ku, Kawasaki 210-0821, Japan*

[6] *Graduate School of Tokyo University of Science, 6-3-1 Niijuku, Katsushika-ku, Tokyo 125-8585, Japan*

† To whom correspondence should be addressed:

Takehiko Tsukahara: ptsuka@lane.iir.titech.ac.jp, Tel, Fax: +81-3-5734-3067

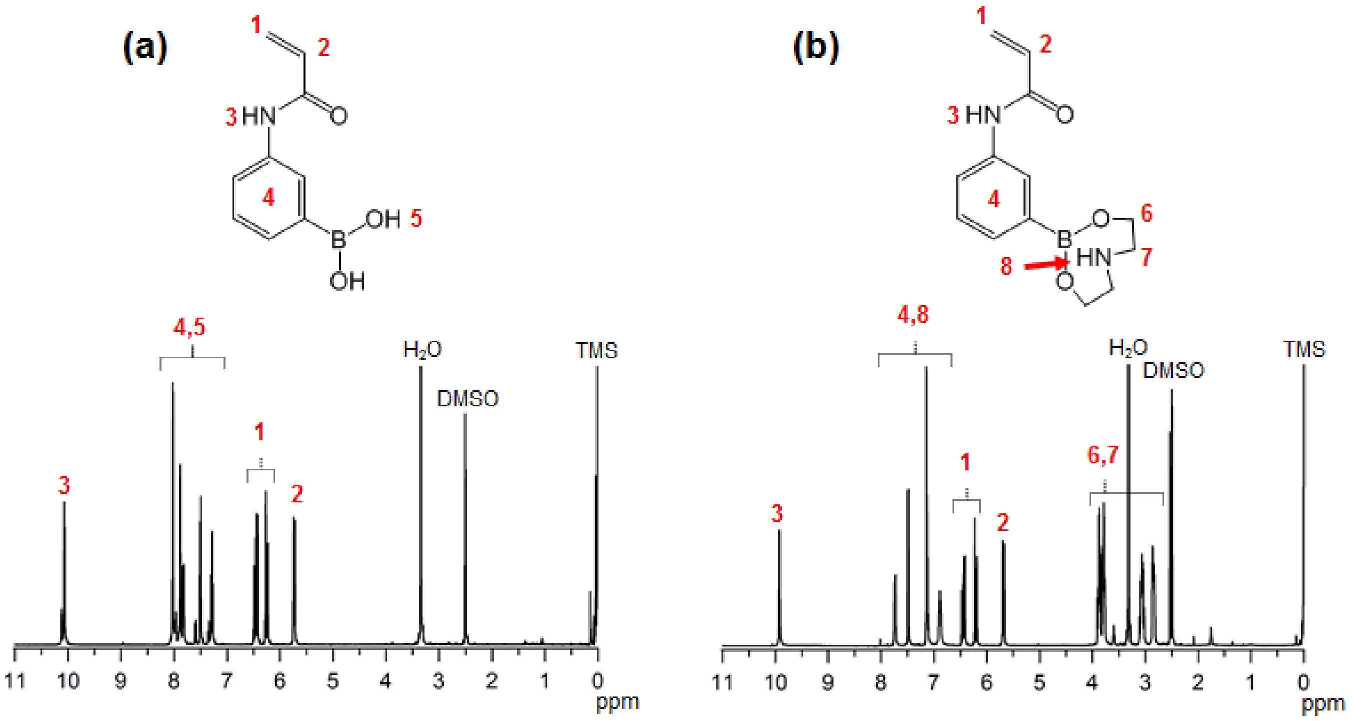

Figure S1. ^{1}H-NMR spectra of (a) PBA monomer and (b) PBA(protected) monomer in DMSO-d_6 solvent at 400MHz (JEOL ECX400P spectrometer) and ambient temperature.

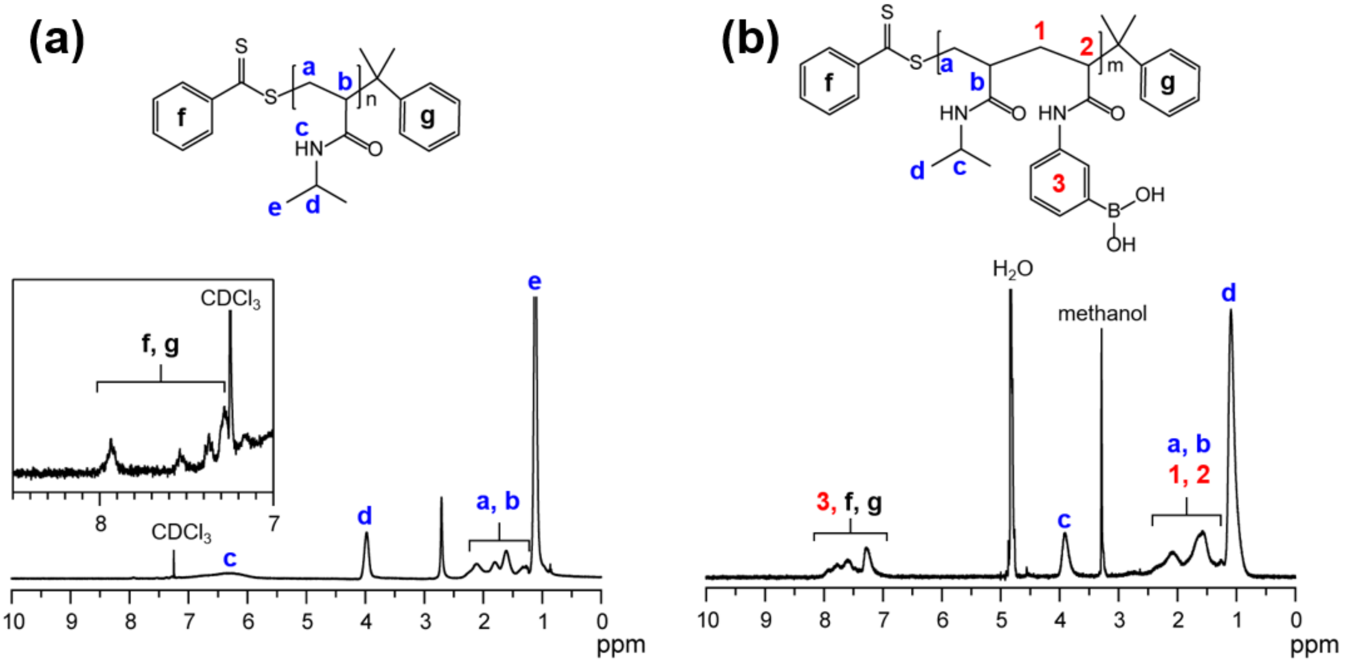

Figure S2. ^{1}H-NMR spectra of (a) poly(NIPAAm) in $CDCl_3$ solvent and (b) poly(NIPAAm-*co*-PBA) in CD_3OD solvent at 400MHz (JEOL ECX400P spectrometer) and 25 °C.

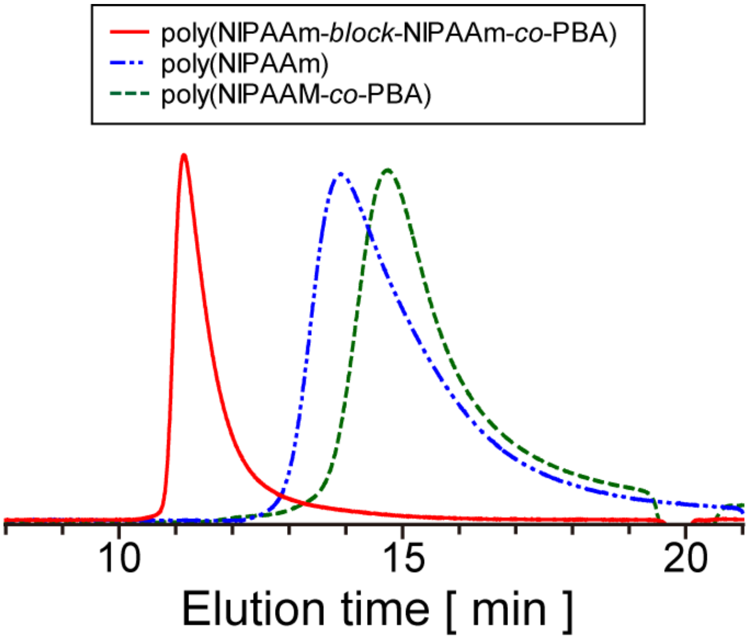

Figure S3. GPC chromatograms of poly(NIPAAm), poly(NIPAAm-*co*-PBA) and poly(NIPAAm-*block*-NIPAAm-*co*-PBA). The GPC measurements were performed by using THF as a mobile phase at 40 °C and the flow rate of 1.0 mL min^{-1}.

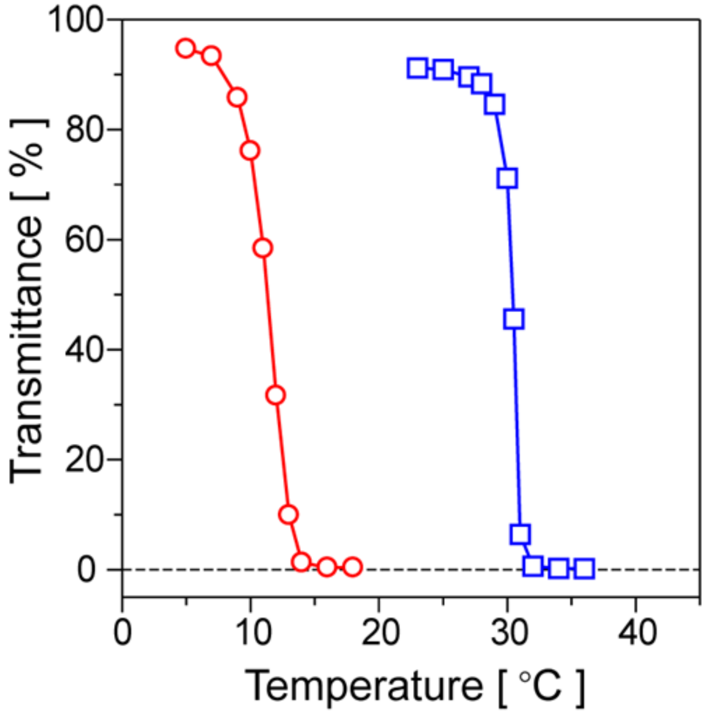

Figure S4. Temperature dependence of optical transmittance of poly(NIPAAm) (open square) and poly(NIPAAm-*co*-PBA) (open circle) dissolved in pure water.

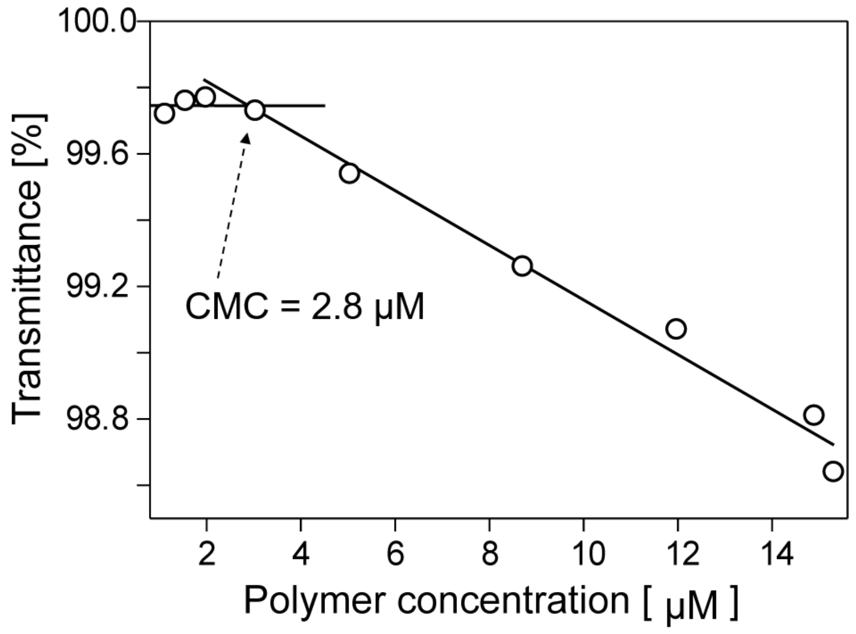

Figure S5. Concentration dependence of optical transmittance of poly(NIPAAm-*block*-NIPAAm-*co*-PBA) dissolved in pure water. The optical transmittance was measured at fixed wavelength of 400 nm. Two straight lines represent the least squares regression lines.
